# Psychometric properties of the Pride in Eating Pathology Scale in a Spanish population

**DOI:** 10.1186/s40337-023-00847-3

**Published:** 2023-07-28

**Authors:** Juan Francisco Rodríguez-Testal, Juana M. Trinidad-Montero, Ángela Rosales Becerra, Cintia Faija, Cristina Senín-Calderón

**Affiliations:** 1 Personality, Evaluation and Psychological Treatment Department, University of Seville, Seville, Spain; 2 Hospital Sant Joan de Déu, Eating Disorders Unit, Barcelona, Spain; 3 Department of Primary Care & Mental Health, University of Liverpool, Liverpool, UK; 4 Department of Psychology, University of Cádiz, Avda. República Árabe Saharaui S/N. Puerto Real, Cádiz, Spain

**Keywords:** Pride, Self-conscious emotion, Eating disorders, Cross-validation, Assessment, General population

## Abstract

**Background:**

In its relation to eating disorders, pride is one of the self-conscious emotions least analyzed, and requires valid and reliable instruments for its measurement. This study aimed to examine the factor structure and the psychometric properties of the Pride in Eating Pathology Scale (PEP-S), in the Spanish general population, as well as between-sex differences in PEP-S scores.

**Methods:**

Of the 1483 participants aged 18 to 34 (*M* = 21.99; *SD* = 3.09), 954 were women (65.2%) and the majority were university students (78.8%). Psychometric properties of the scale were tested in a cross-sectional design using cross-validation, i.e., exploratory and confirmatory factor analysis, and estimation of invariance (sex).

**Results:**

The four-factor structure found was similar to the original scale with invariance across sex and internal consistency (ordinal alpha .99) and stability (.85). Evidence of convergent validity and differences between sexes were found. Specifically, women scored higher on all the factors, including the healthier sense of pride.

**Conclusions:**

The PEP-S scale is an instrument with evidence of validity and reliability in the Spanish population. Although it still has to be tested in a clinical population, it constitutes a promising instrument for the evaluation of the self-conscious emotion, pride.

**Supplementary Information:**

The online version contains supplementary material available at 10.1186/s40337-023-00847-3.

## Abstract (plain language summary)

The study of eating disorders emphasizes the role of certain emotions, such as pride, for example, in achieving a low weight or controlling eating behavior. We propose the Spanish validation of the Pride in Eating Pathology Scale (PEP-S), developed by Faija et al. (2017), to measure this type of complex or self-conscious emotions. This article confirms the usefulness of the PEP-S in the context of the general population, different from the context of the clinical population in which it was validated, which implies a generalization of its possibilities. In addition, as a novelty, it includes male participants, who are also subjected to the social pressure on body and appearance. This validation of the PEP-S was carried out in a large sample of men and women aged 18 to 34, a time of life when concerns about the body and eating behavior acquire importance. It is worth special mention that the results of its designers were confirmed, with psychometric indicators guaranteeing that the instrument can be used with consistency of measurement (reliability) and usefulness (validity) in the general population. Men and women respond to the instrument the same, that is, they understand the scale the same way. The scores of women on the PEP-S are higher in terms of pride about the body and eating behavior. Future studies should test these promising results in a clinical population.

**Supplementary Information:**

The online version contains supplementary material available at 10.1186/s40337-023-00847-3.

## Introduction

Emotions act as motivational factors that influence personal and social adaptation [1]. In addition to the basic emotions, complex or self-conscious emotions [2], such as shame, guilt and pride affect personal and behavioral self-evaluation [3], as well as evaluation of the image projected toward others [4]. In spite of their impact on identity and behavior, or more generally speaking, subjective wellbeing, research on these self-conscious emotions is still very incipient. They often focus on their relationship with depression or anxiety [5] or on bipolar disorders [6], on their identification through language or facial expression [7, 8], or on isolating and analyzing some of them, such as guilt and shame, because of their connection to anxiety [9], or with psychotic disorders (shame) [10], to name a few.

Self-conscious emotions have recently been studied as related to Eating Disorders [11]. Difficulties in their regulation influence one’s system of beliefs, self-evaluation and social attributions (Their own and others’) [12]. Thus, shame, guilt or pride have been identified as precursor variables that maintain eating disorders, and the role of each can be differentiated [13–16]. Pride, however, has received less attention in research [13, 17]. In the university population, for example, pride is related to presence of indicators of EDs and of morbid exercise behavior [18]. In another study with women in the general population, it was found that women at risk of EDs had high scores in shame or guilt, but low scores in pride, these relationships mediated by anxiety and depression [19]. It may be said that the satisfaction from gaining control of food intake and nearing the “ideal” image generates a highly positive reinforcement that favors perpetuation of restrictive behaviors, and are emotionally experienced as pride [14]. Pride, as a self-conscious emotion, motivates people to perform behaviors that are socially valued, representing success and favoring positive self-evaluation [20]. The achievement of success may be transferred to several different behaviors in ED including overcoming the desire to eat, limiting or avoiding intake of “forbidden foods”, enduring intense physical activity and nearing the patients closer to a distorted body image which they are highly involved with, for example, by strengthening self-esteem and perceived control of behavior [17]. The importance of social recognition is related to the low self-esteem and body dissatisfaction characteristic of EDs [21].

In the literature there are only a few validated instruments that evaluate these complex emotions and specifically pride. We could mention the *Body and Appearance Self-Conscious Emotions Scale* (BASES [22]), which is adapted to the Spanish population [23], and analyzes shame, guilt, authentic pride and hubristic pride (excessive, arrogant, pompous, conceited pride); and the Test of Self-Conscious Affect (TOSCA [24]), which analyzes guilt, shame, detachment and alpha (in self) and beta (in behavior) pride. This last facet showed low internal consistency in its design, and also in later validations, and research has not recommended its use [22–24].

Unlike the above-mentioned instruments, the *Pride in Eating Pathology Scale* (PEP-S) [25] has been specifically developed to measure pride in Eating Disorders. The PEP-S is comprised of a total evaluation of pride and four specific dimensions: One is linked to the feeling of pride emerging from the satisfaction of managing to restrict food intake, losing weight and being thin (“Pride in weight loss, food control, and thinness”), two dimensions on external pride due to the perception of pride stemming from an external validation produced by appreciation of weight loss (“Pride in outperforming others and social recognition”, and “Pride in capturing other people’s attention due to extreme thinness”), plus a dimension that can be considered a protective factor in the sense of healthy behaviors and habits (“Pride in healthy weight and healthy eating”), which is hypothesized to become more relevant in the recovery stage of an ED [13].

In the present study we translated the PEP-S into Spanish and tested its psychometric properties when adapted to the Spanish-speaking population [25] This would enable the possibility of applying this instrument both in the scientific, and clinical setting, and would be useful for future development of models of the origin and maintenance of ED with regard to the self-conscious emotion of pride.

The study of evaluation of pride has mostly focused on women with ED. However, social changes and overexposure of physical image in Western culture have caused these alterations to increase in the male population [25, 26]. Studies on how weight control, pressure on muscle tone, or food control and restriction behaviors affect the male population are essential to understanding eating behaviors in this population and those studies are currently limited [27]. We think that although both men and women show these disorders, the PEP-S can be proposed as an instrument capable of assessing the role of pride in EDs. This would enable verification not only of the scores, but also whether men and women have a differential understanding of pride in relation to the body and eating behaviors, as confirmed for shame and guilt by the TOSCA [26].

In view of all of the above, this preliminary study proposed validation of the PEP-S scale with the following specific objectives: (1) Examine the factor structure of the PEP-S in a sample from the Spanish general population (evidence of internal validity); (2) Analyze the invariance of measurement of the PEP-S across sex; (3) Estimate the reliability of the PEP-S scores and check its measurement stability; (4) Compare the evidence of validity of the PEP-S to other scales (convergent validity); (5) Compare the differences between sexes in the scale’s dimensions and its total score.

## Method

### Participants

The original sample of 1705 participants was selected by accessibility and snowball sampling of the general population. After filtering (*n* = 242) by age and eliminating those under 18 or over 35, and/or whose answers on a control scale (EPI Sincerity) were < 5 points) [28], the final sample was comprised of 1483 participants aged 18 to 34 (*M* = 21.99; *SD* = 3.09), of whom 509 were men (34.8%) and 954 were women (65.2%). The participants were mostly single (93.9%), the average Social Class Index [29] was 36.80 (*SD* = 17.39; middle class; range 11 to 77), with a large proportion of undergraduate students (78.8%) or higher education (55.2%), 79.7% had no history of a psychological disorder, 9.2% were currently under psychological or psychiatric treatment, and 5% were taking psychotropic medication (anxiolytics and/or antidepressants). Sample characteristics are presented in Table 1.

**Table 1:** Sample characteristics (*N* = 1483)

Gender		Age	Social class index
Female	954 (65.2%)	*M* = 21.99*(SD* = 3.09)	*M* = 36.80 (*SD* = 17.39)
Male	509 (34.8%)	min = 18; max = 34	min = 11; max = 77

Marital status		Level of education		Occupation	
Single	1329 (90.8%)	Compulsory	33 (2.2%)	Students	1168 (78.8%)
Married	88 (6.0%)	Upper-secondary	632 (42.6%)	Employed	234 (15.7%)
Divorced	1 (0.1%)	Higher education	818 (55.2%)	Unemployed	81 (5.5%)

History psychological problems		Psychological treatment		Pharmacological treatment	
No	1166 (79.7%)	No	1329 (90.8%)	No	1390 (95%)
Yes	297 (20.3%)	Yes	134 (9.2%)	Yes	73 (5%)

### Instruments

#### Basic sociodemographic information record sheet (by authors)

These records were used to collect sex, date of birth, marital status, occupation, education, history of psychological problems, and psychological treatment/medication.

#### Eysenck personality inventory subscale (EPI sincerity)

Eysenck Personality Inventory Subscale [28] with nine true/false items that identify dishonest answers. Participants who scored below five were excluded.

#### Pride in Eating Pathology Scale (PEP-S)

This is a self-report questionnaire assessing pride related to eating disorder behaviors [30]. Its 60 items evaluate pride on a Likert-type scale of 1 (Strongly Disagree) to 7 (Strongly Agree). Four factors were found in the construction of the instrument: Factor 1. Pride in weight loss, food control, and thinness (e.g., “I would feel proud if I fit into a smaller clothing size”); Factor 2. Pride in healthy weight and healthy eating (e.g., “I feel proud of what I have achieved in the way my body looks”); Factor 3. Pride in outperforming others and social recognition (e.g., “Eating less than others makes me feel proud”); Factor 4. Pride in capturing other people’s attention due to extreme thinness (e.g., “When other people comment on my extreme thinness, I feel a sense of pride”). The scale also provides a Total score which includes Factors 1, 3 and 4. The internal consistency of this instrument was α = 0.98, 0.88, 0.96, a 0.90, on Factors 1 to 4, respectively. The PEP-S is described in the appendix of the study.

#### Body and appearance self-conscious emotions scale (BASES)

This instrument is a self-report consisting of 15 items (Spanish version by Alcaraz-Ibáñez and Sicilia [23] and 16 in the original version [22], on a 5-point Likert scale, ranging from 1 (Never) to 5 (Always). It has four factors (four items per factor): authentic pride (focused on achievement by controlled, adaptive behaviors; e.g., “I am proud of the effort I make on maintaining my appearance”); hubristic pride (focused on grandiosity and superiority over others, maladaptive, e.g., “I am proud that I am more attractive than others”); guilt, and shame. Internal consistency of the original version was α = 0.93 authentic pride, 0.91 hubristic pride, 0.91 guilt, and 0.85 shame. For the Spanish validation it was α = 0.89 authentic pride, 0.90 hubristic pride, 0.89 guilt (only three items), and 0.89 shame. In this study, α = 0.90 authentic pride, 0.89 hubristic pride, 0.89 guilt, and 0.90 shame.

#### Depression, anxiety and stress scales (DASS-21)

This self-report has three subscales with seven items for evaluating depression, anxiety and stress during the past week [31] (Spanish version by Fonseca-Pedrero et al. [32]). The frequency the items fit the person evaluated is rated from 0 (*Did not apply to me at all*) to 3 (*Applied to me very much or much of the time*). The original version had adequate internal consistency. In the Spanish validation, the total α = 0.90, and 0.80, 0.73 and 0.81, for depression, anxiety and stress, respectively.

### Procedure

Permission was received to use the authorized version of the PEP-S in Spanish. The study had a cross-sectional ex post facto design. The participants were recruited by accessibility, not randomly (e.g., stratified sampling in proportion to their relative size in the population), and by snowball sampling. Students in upper degree courses in Psychology were offered the possibility of participating voluntarily, for which they were given academic credit (an alternative procedure for academic credit was available to those who did not participate in the study). Students had to find four other participants from their setting (friends, partner and/or relatives) who wished to volunteer for the study (snowball sampling, to widen the diversity of participants), and these participants filled in the tests with supervision by the students participating in the study (so that the original instructions remain unchanged). Inclusion criteria were that they be of legal age, a native Spanish speaker, and follow the instructions for completing the tests (adequate light and acoustic isolation conditions). The exclusion criteria were under 18 and over 35 years of age (to avoid wide data dispersion) and scoring below five on the EPI sincerity scale [28]. Participants undergoing psychological or psychiatric treatment were not excluded, as they represented a very small percentage of the sample. It was estimated that for an instrument of approximately 10 ordinal scale indicators (based on the PEP-S scale), at a ratio of 20:1 (N:q) (Kyriazos, 2018), at least 600 participants were necessary. Data were collected online based on a link the participants received on their electronic device (computer, tablet and/or cell phone), in a test–retest procedure (one month between PEP-S measurements), after ensuring them confidentiality and receiving their informed consent. There was no time limit for completing the battery of tests (estimated at about 40 min) and participants were informed that honesty would be controlled for. Precise instructions were given for each test, and when they were finished, they had to move on to the next test and could not go back. As the questions had a closed format, lost values were controlled for. The study followed the precepts of the Helsinki Declaration and was approved by the Bioethical Committee of the Andalusian Government.

### Data analysis

Descriptive statistics of the measurements and participant characteristics were reported. Most of the analyses were for cross-validation of the PEP-S following the original publication [25]. For the first objective, the sample of women was divided into two halves (as in the original validation), and it was confirmed that both samples were equivalent in age, social class and total PEP-S score (see Additional file 1: Table 1). Half of the sample was used for exploratory factor analysis to find the internal scale structure, and the other half for confirmatory factor analysis which tested two models. The Diagonally Weighted Least Squares (WDLS) method, which is a robust estimator for modelling ordinal data, is recommended for use when multivariate normality is violated [33]. Consequently, this was used with oblique rotation (direct oblimin) for both the EFA and CFA. Model fit was evaluated with the comparative fit index (CFI) and non-normed fit index (NNFI), which must be > 0.90 [34]. The root mean square error of approximation (RMSEA) and its 90% confidence interval (CI) were calculated, which for a good fit must be < 0.08, values from 0.08 to 0.10 indicate a mediocre fit and > 0.10 indicates clear misfit [33, 34]. Akaike’s Information Criterion (AIC), which can evaluate model parsimony, where the model that best fits is the one that has the lowest values, was also calculated.

For the second objective, a multi-group CFA was estimated to test measurement invariance across sex in a series of hierarchical steps in which increasingly stringent restrictions were placed on the parameters to be estimated. Configural invariance was tested by estimating the same factor structure simultaneously in men and women without imposing restrictions between the groups in estimating the parameters. To test metric invariance, factor loadings were constrained to be equal across the groups. Finally, to test scalar invariance, restrictions were constrained to be equivalent on factor loadings and thresholds. The comparisons of model fit were evaluated with Chen’s criterion [35] through the levels of invariance (configural vs. metric; metric vs. scalar). The hypothesis of invariance was accepted if ΔCFI < 0.01 and ΔRMSEA < 0.015 [35].

For the third objective, the reliability of the PEP-S scores was found using the ordinal alpha and ordinal omega for the total scale and for its factors. Test–retest reliability was assessed with Spearman’s correlation, the discrimination indices and intraclass correlation coefficient.

For the fourth objective, evidence of convergent validity was found with Spearman’s correlations.

Finally, for the fifth objective, a Student’s *t*-test between sexes was done for the PEP-S dimensions and total score, and the effect size was found using Cohen’s *d*. Statistical analyses were performed with SPSS ver 0.24, Lisrel ver. 8.7, Jasp ver. 0.16.3 and Jamovi ver. 2.3.18.0.

## Results

### Preliminary analyses

The sample of women (*n* = 954) was divided into two halves. Both samples were equivalent in age, social class index and total PEP-S score (Additional file 1: Table A1).

### Evidence of validity based on the internal structure (Objective 1)

The Exploratory Factor Analysis (EFA) was calculated with the first half of the sample of women (*n* = 472) using Robust Diagonally Weighted Least Squares (RDWLS) and Robust Promin [36]. The parallel analysis was initially favorable for a four-factor structure (Additional file 1: Table A2). Descriptive statistics were analyzed for skewness (> 1 in 21 of the items, so somewhat more than a third of the items showed agreement with the high scores in pride) and kurtosis (clearly high in 9 items). The Mardia test was statistically significant for kurtosis, but not skewness, indicating that the assumption of multivariate normality was not met (128.549, *p* < 0.001). The KMO (0.974) and Bartlett’s test (5182.7, *df* = 1770, *p* < 0.0.001; matrix determinant χ^2^(1536) = 31,463.305, *p* < 0.001) suggested adequate results. The Measure of Sampling Adequacy (MSA) found no values under 0.50, so exclusion was not recommended for any item. The communalities were all above 0.30 on the items, except items 25 (0.193) and 27 (0.291). Eigenvalues explained variance of 74.96%, Factor 1: 34.70 (57.83%), Factor 2: 5.81 (9.69%), Factor 3: 2.93 (4.87%), Factor 4: 1.21 (2.02%). The robust goodness-of-fit indices found were the Root Mean Square Error of Approximation (RMSEA) = 0.039 (CI 0.010, 0.050), the Non-Normed Fit Index (NNFI) = 0.999, and the Comparative Fit Index (CFI) = 0.999, which are adequate [34]. Factor loadings of items and factors were reported in the Additional file 1.

The proposed structure would be more safely composed of three factors, with doubts about Items 25 and 27 (low communality). Factor 1, Pride in weight loss, food control. and thinness: 1, 4, 5, 9, 10, 11, 17, 18, 19, 22, 25, 26, 28, 29, 36, 39, 40, 42, 46, 49, 51, 53, 59, 60; Factor 2. Pride in outperforming others and social recognition: 6, 7, 8, 12, 14, 15, 20, 21, 23, 24, 31, 32, 33, 35, 37, 41, 43, 45, 47, 48, 50, 54, 55, 56. Factor 3. Pride in healthy weight and healthy eating: 13, 16, 27, 30, 34, 38, 44, 52, 57, 58.

Factor 4 in the original test, Pride in capturing other people’s attention due to extreme thinness, might not be taken into account as being comprised of only two items (2 and 3).

Confirmatory Factor Analysis (CFA) was performed with the second half of the sample of women (*n* = 482) using DWLS with the asymptotic covariance matrix. The first model (Model 1) analyzed tested the three-factor structure found after performing CFA, and the second model (Model 2) analyzed the four-factor structure found in the original scale published by Faija et al. [25] (in Additional File 1). Table 2 presents the goodness-of-fit indicators for both models. Although the model that analyzed the factor structure found in the EFA had adequate goodness-of-fit indicators, the model by Faija, et al. [25] fit the data better, mainly CFI, NNFI, and RMSEA (Table 2). Furthermore, the AIC was lower for Model 2 in later analyses.

**Table 2:** Fit indices of the PEP-S scale

Model	*χ*^2^ Satorra–Bentler	*df*	CFI	NNFI	SRMR	RMSEA [90% CI]	AIC
Model 1:	21,695.83*	1709	.93	.93	.09	.10 [.10, .10]	9875.14
Model 2:	8653.22*	1704	.98	.98	.09	.09 [.09, .09]	8905.22

CFI, comparative fit index; NNFI, non-normed fit index; SRMR, root mean square residual; RMSEA, root mean square error of approximation; AIC, Akaike’s information criterion

*p < .01. Model 1 from EFA; Model 2: Original Factor structure (Faija et al. [25])

The four-factor model (Factor 1. Pride in weight loss, food control, and thinness, WL; Factor 2. Pride in healthy weight and healthy eating, HW; Factor 3. Pride in outperforming others and social recognition, SR; Factor 4, Pride in capturing other people’s attention due to extreme thinness, CA) had very low factor loadings for Items 2, 3 and 25 with saturations of 0.28, 0.12 y 0.21, respectively. These three items loaded on Factor 3. The rest of the factor loadings varied from 0.65 (Item 27, Factor 3) to 0.97 (Item 30, Factor 3). The items with the lowest percentage of explained variance were 2, 3 and 25 (*r*^2^ = 0.08, *r*^2^ = 0.01 and *r*^2^ = 0.04), and these items also showed high residual variances of 0.94, 0.99 and 0.97, respectively. These results suggest that the three items mentioned are not very reliable. The items with the highest percentage of explained variance were 29 (*r*^2^ = 0.94, Factor 1), 26 (*r*^2^ = 0.93, Factor 4), 46 (*r*^2^ = 92, Factor 1) and 53 (*r*^2^ = 0.91, Factor 1). The correlations between the factors were moderate (Factor 2–3 *r* = 0.44; Factor 3–4 *r* = 0.42 and Factor 1–3 *r* = 0.50) to high (Factor 1–2 *r* = 0.85; Factor 2–4 = 0.88; Factor 1–4 = 0.82).

### Invariance of PEP-S measurement across sex (Objective 2)

Analyses of invariance across sex were performed on the four-factor model found by their originators [25]. Before evaluating the invariance with the groups, the model was estimated separately in each sample (women and men). The model fit was reasonable, although for men (*n* = 509), the RMSEA was slightly higher than recommended. The configural invariance of the model adjusted to the data well, which suggests that the four-factor structure is equivalent in both groups. As shown in Table 3, when factor loadings were constrained to be equal to test metric invariance, the model showed no misfit (ΔCFI < 0.01 and ΔRMSEA < 0.15), which suggests that the magnitude of the factor loadings in the two groups was statistically equivalent. On the second level of invariance (scalar), when the factor loadings and item thresholds were constrained to be equal, the model remained stable, which suggests that the scores on latent variables in the two groups can be compared. These results support invariance of PEP-S measurement across sex.

**Table 3:** Invariance of measurement of the PEP-S across gender

Model	SB*χ*^2^	*df*	CFI	NNFI	ΔCFI	RMSEA [90% CI]	ΔRMSEA
Men	12,318.58*	1704	.96	.96		.11 [.11, .11]	
Women	15,127.06*	1704	.98	.98		.09 [.09, .09]	
Configural invariance	27,489.94*	3408	.97	.97		.09 [.09, .09]	
Metric invariance	28,358.77*	3464	.97	.97	.00	.09 [.09, .10]	.00
Scalar invariance	26,748.88*	3524	.97	.97	.00	.10 [.09, .10]	.01

CFI, comparative fit index; NNFI, non-normed fit index; RMSEA, root mean square error of approximation

### Reliability estimation of PEP-S scores (Objective 3)

Internal consistency of the items in the four factors, as well as the total scale, were excellent for the complete sample (*N* = 1463). Test–retest reliability with a time span of one month was favorable for the total measure and Factor 1 (WL), but the retest reliability of Factor 2 (HW) did not comply with the indicator > 0.7 [37]. The intraclass correlation indices were optimum [38]: 0.979 for the original total measurement [CI 95% 0.977–0.980] and 0.980 for the retest [CI 95% 0.978–0.982]. The discrimination indices showed a very favorable item-total relationship for Factors 1 (WL) and 3 (SR), but less favorable for Factor 2 (HW). Table 4 gives the reliability of the scores found with the ordinal alpha and ordinal omega.

**Table 4:** Reliability of the PEP-S scores

PEP-S	Ordinal alpha	Ordinal omega	Discrimination index	Test retest (*r*)^a^
PEP-S-WL^a^	.99	.99	From .60 to .91	.861**
PEP-S-HW	.93	.91	From .38 to .77	.689**
PEP-S-SR	.99	.98	From .63 to .84	.796**
PEP-S-CA	.87	.89	From .52 to .67	.710**
PEP-S-Total	.99	.99	–	.856**

Original scale by Faija et al. [25]. Factor 1. Pride in weight loss. food control. and thinness (WL); Factor 2. Pride in healthy weight and healthy eating (HW). Factor 3. Pride in outperforming others and social recognition (SR); Factor 4, Pride in capturing other people’s attention due to extreme thinness (CA)

^a^Spearman’s correlations ** *p* < .001

### Evidence of validity with respect to other scales (Objective 4)

Table 5 presents the evidence of validity compared to other scales. The relationship of Factor 2 with the BASES authentic pride subscale should be emphasized. The relationship of the pathological factors in the PEP-S (1, 3 and 4) with the DASS-21 Depression, Anxiety and Stress scale should also be highlighted. PEP-S Factor 3 was strongly related to the DASS-21 depression, anxiety and stress scale. Finally, the relationship of the PEP-S clinical index (Factors 1, 3 and 4) with shame and guilt (BASES) and the Depression, Anxiety and Stress Scale (DASS-21) should be underlined. However, most of the correlations, although significant, are low.

**Table 5:** Signs of convergent validity with other scales (*N* = 1483)

	BASES				DASS-21		
	BP	BHP	BS	BG	DD	DA	DS
PEP-S-WL^a^	.172**	.072	.527**	.554**	.206**	.194**	.203**
PEP-S-HW	.517**	.263**	.062	.118**	-.008	.049	.115**
PEP-S-SR	.227**	.183**	.460**	.412**	.259**	.238**	.250**
PEP-S-CA	.101*	.097*	.446**	.375**	.191**	.180**	.181**
PEP-S-Total	.252**	.147**	.504**	.504**	.204**	.200**	.223**
PEP-S-Clin	.194**	.117**	.538**	.534**	.228**	.212**	.223**
*M *(*SD*)	6.02 (5.06)	10.31 (4.03)	9.12 (4.12)	8.35 (3.47)	11.51 (4.29)	5.54 (5.02)	8.41 (5.06)

^a^Spearman’s correlations. * *p* < .05; ** *p* < .01. Note: Original scale by Faija et al. [25]. Factor 1. Pride in weight loss, food control, and thinness (WL); Factor 2. Pride in healthy weight and healthy eating (HW). Factor 3. Pride in outperforming others and social recognition (SR); Factor 4, Pride in capturing other people’s attention due to extreme thinness (CA); PEP-S-Clin, clinical score = (WL + SR + CA)/3; BP (BASES- pride); BHP (BASES-hubristic pride); BS (BASES-shame); BG (BASES-guilt); DD (DASS-21-depression); DA (DASS-21-anxiety); DS (DASS-21-stress)

### Comparison of mean PEP-S scores across sex (Objective 5)

A Student’s *t*-test was done for the effect of sex on the PEP-S factors and total score. Statistically significant differences were found in both total score and on three factors, except for “Pride in outperforming others and social recognition” (trending result). As shown in Table 6, women scored higher than men on all factors. However, the effect size was small.

**Table 6:** Comparison between genders of means on the PEP-S dimensions and total score

PEP-S	*M* (*SD*) Men (*n* = 509)	*M* (*SD*) Women (*n* = 954)	*t *(*gl*)	*p*	Cohen’s *d*
PEP-S-WL^a^	81.67 (42.81)	94.35 (48.53)	5.15 (1154.14)	< .001	.27
PEP-S-HW	46.90 (15.98)	49.81 (14.39)	3.54 (948.29)	< .001	.19
PEP-S-SR	36.67 (21.42)	39.07 (24.96)	1.92 (1179.96)	.055	.10
PEP-S-CA	5.62 (3.67)	6.42 (4.11)	3.64 (1141.12)	< .001	.20
PEP-S-Total	142.65 (70.72)	160.03 (81.46)	4.06 (1169.46)	< .001	.22

^a^Original scale by Faija et al. [25]. Factor 1. Pride in weight loss, food control, and thinness (PEP-S WL); Factor 2. Pride in healthy weight and healthy eating (PEP-S HW). Factor 3. Pride in outperforming others and social recognition (PEP-S SR); Factor 4, Pride in capturing other people’s attention due to extreme thinness (PEP-S CA)

## Discussion

This study showed that pride is of interest as a self-conscious emotion which has been analyzed in the context of EDs. The general objective was the validation of the PEP-S [25] in the Spanish context (although it was also compared to different measurements used for the original validation). Our study shows that PEP-S can measure the role of pride in ED and can be applied to the general population [37] in screening for early ED detection. It could also be useful for follow-up of clinical cases, and longitudinal research designs that aim to separate the role of the different components of pride in ED.

As the first objective, the PEP-S factor structure was examined in a sample from the Spanish general population. It was found to be similar to the original scale, and was corroborated in a four-factor model identical to the one found by Faija et al. [25]. Therefore, although the results of the confirmatory analysis endorsed the original structure, it was further considered important to maintain the theoretical conception which was the basis of the instrument’s construction. This is relevant, because there could have been cultural differences in the Spanish adaptation, as there have been in other instruments. However, this was not the case. Three factors in this structure are considered more pathological (pride in weight loss-thinness, outperforming others and social recognition, and pride in capturing other people’s attention due to extreme thinness), as well as a more positive or adaptive factor (pride in healthy behavior that entails a more positive self-evaluation). The first three factors synthesize one of the essential components of ED, since the way for them to improve their self-esteem and feel proud, may go through resisting desires, for example, for food, escaping from the control of others, or appearing better than others because of having made this effort and achieved control [14]. In short, because pride is related to control (thinness), it implies social achievement, and recognition raises self-esteem.

The second objective verified the invariance of measurement across sex, as although the instrument was originally developed for women, it showed considerable invariance (stability) for men as well. Men are also affected by beauty standards, and this could be influential in the increase in ED in this population [26, 39]. Our findings support invariance in measurement across sex, which suggests that the mean scores of men and women on the PEP-S factors may be compared: the latent dimensions measure the same constructs the same way in both sexes. This represents an additional contribution to the validation of the original instrument, and although men and women may manifest ED differentially, the PEP-S enables the role of pride to be evaluated by how it is answered regardless of sex. There is a paucity of work analyzing pride in men in the general population. Some contributions have studied EDs in men, but only in very specific populations (i.e., athletes) [40]. Therefore, this study also contributes the novelty of considering pride in men, given the usual tendency to exclude them in the study of EDs [41].

The third objective estimated the reliability of the scores and their retest stability. Both alpha and omega values showed that the instruments’ scores were clearly reliable, and equivalent to the original version [25]. Stability standards for the total scale and its factors were met, except for Factor 2 (evaluating pride from a more favorable perspective), which was at the limit at the one-month follow-up. This might be explained by the limited number of items composing this factor (i.e., only three items).

The fourth objective was to study evidence of convergent validity with other measures. The PEP-S and BASES self-conscious emotion factors [22] were compared. The correlations were not very strong, except for Factor 2 (more positive view of pride), indicating that the two scales may measure different aspects of pride. Specifically, one of the advantages of the PEP-S is that three of its factors evaluate a clearly pathological conception of pride, which could contribute to the study of why pride has been attributed a role, whether as protection or risk, in ED development or maintenance [42]. Furthermore, the PEP-S scale focuses on behaviors and consequences (or goals with respect to eating behavior) that are very specific to EDs, while the BASES scale factors refer to very general aspects of appearance. In the general population, the PEP-S and its factors (Factors 1, 3 and 4) were related to the BASES shame and guilt subscales [43], so pride can be understood either negatively or in combination with other self-conscious emotions. Therefore, the PEP-S may highlight the difficulty in differentiating some self-conscious emotions. In populations with ED or body image disturbances, pride may be related more to self-esteem motivated by success. The PEP-S was not strongly related to emotional symptoms (DASS-21; [31]) for the same reason as above. In other words, in the general population, the symptoms are less prominent and less related to the items indicating typical ED behaviors. In any case, Factor 3 (Pride in outperforming others and social recognition) was related to emotional measurements.

The last objective aimed at individuating any sex differences in the instrument [44]. Women scored higher on all the factors, including the healthier sense of pride, however, this difference does not appear to be significant in pride in outperforming others and social recognition. Some recent findings show more equalization between males and females in adolescent anorexia nervosa and a wider dispersion in bulimia nervosa [39]. Some of our findings regarding body image are therefore tentative. On the one hand, the differences found with respect to females could be attenuated because of a greater presence of males in relation to body image problems (as shown in one of the PEP-S factors). On the other hand, this equalization occurs from adolescence onwards [39], which indicates long-lasting patterns related to these difficulties, a point that requires further research.

Summarizing, the PEP-S scale is an instrument with robust evidence of validity and reliability which can be applied to the Spanish general population. Its internal structure and other indicators must still be tested in a clinical population to corroborate the original proposal. The importance of developing longitudinal studies has also been highlighted [43, 44], especially in shame-shame [42] and/or shame-pride cycles relevant to the genesis and maintenance of ED [14, 44], which would make it possible to see the long-term stability of the instrument.

In addition to a longitudinal perspective, it should be confirmed that pride has a mediating role in the genesis and maintenance of ED [14]. The results of this study, although done in the general population, suggest that pathological pride (Factors 1, 3, 4) is related to shame and guilt, while more beneficial or constructive pride is not (Factor 2). High scores in pathological and egosyntonic pride are to be expected in the clinical population, loading on success sustained by control and restriction, success that shows superiority over others, or that enables one to value the scope of this control. That is, pride that somehow involves more positive self-evaluation distanced from fear of weight gain, fat, and compensatory behaviors [45]. This would suggest that high scores in pride would be related to more control-focused EDs as well as obsessiveness, perfectionism and rigidity (especially in anorexia nervosa) [46]. Future research should determine whether pride is a trait characteristic (possibly linked to the need for control/dominance, obsessiveness, and low self-esteem), or a state involving loss of control (shame) or gaining control by being successful in food restriction (pride) [16, 45], for example. This is relevant for adequate orientation of a treatment that contributes to the management of negative emotions, a better response to the perception of loss of control and balanced self-esteem.

This study had some limitations that should be taken into account. In the first place, it had a cross-sectional design, which did not comprise random selection applied to the general population, and which therefore affects generalization of the results. Furthermore, the participants came mainly from a university population, which is another limitation that should be kept in mind. In addition, the choice of students by other students suggests a potential risk of bias. A minority of the participants may also have shown signs of a psychological/psychiatric disturbance, and it cannot be ruled out that some might have had an ED. However, the sample was large, and was dealt with following a procedure that validated the internal structure of the PEP-S. In the second place, demographic data were collected differentiating between men and women. It is pertinent for the analysis of sex diversity to be taken into account in further studies. Thirdly, there was no follow-up on the scores, nor was a prospective perspective taken, so that some assumptions about whether the instrument can be used for follow-ups and treatments require more evidence. Fourth, the study essentially compared the original PEP-S study (although not all measures), but did not specifically analyze cultural differences in the instrument. Nevertheless, in its adaptation to Spanish, the authors revised its characteristics, and independently confirmed the suitability of this version of the instrument. Fifth, the study included a sample of male participants. Although the instrument was not designed for that, the results showed clear invariance. However, more testing should be done keeping this in mind for its use with samples of men, for example, with ED. Finally, the study was designed to collect data from the general population, although no other instruments related to ED were used. It was designed as an exploratory study, and for its later use to study alterations in eating behavior.

## Conclusions

The PEP-S scale is an instrument with evidence of validity and reliability in the Spanish general population. The PEP-S can characterize the role of pride in ED and can be used with a general population [37] in screening for early detection, and could prove useful in following a treatment that has begun. It would also facilitate more complex, longitudinal research designs that separate the role of different components of pride in ED. It was also demonstrated that men and women respond similarly to the instrument, regardless of the differences in the total scores. This novelty may allow us to study the presence of pride in relation to EDs in the male population, as has been done with women. Although it still has to be tested in a clinical population, it constitutes a promising instrument.

## Supplementary Information

**Additional file 1**. Appendix A, Supplementary tables.

## Data Availability

The datasets used and/or analyzed during the current study are available from the corresponding author upon reasonable request.

## Abbreviations

BASES: Body and appearance self-conscious emotions scale
DASS-21: Depression, anxiety and stress scales
ED: Eating disorder
EPI: Sincerity Eysenck personality inventory subscale
PEP-S: Pride in Eating Pathology Scale
TOSCA: Test of self-conscious affect
