# Supplementary material for: Psychometric properties of the Pride in Eating Pathology Scale in a Spanish population
Source: J Eat Disord. 2023 Jul 28;11:124. doi: 10.1186/s40337-023-00847-3 (PMC10386289; doi:10.1186/s40337-023-00847-3)
Supplement: Supplementary file 1 — Additional file 1. Appendix A, Supplementary tables. [file 40337_2023_847_MOESM1_ESM.docx]

**Appendix A**

Original Pride in Eating Pathology Scale (PEP-S). Faija et al. [25]

1) I would feel pride if I fit into a smaller size of clothing. F1

2) I feel proud of what I have achieved in the way my body looks. F2

3) I feel pride because I look fit. F2

4) I would feel more pride if I am skinny. F1

5) I feel proud of myself when the numbers on the scales go down. F1

6) Eating less than others makes me feel pride. F3

7) I experience a sense of pride when I don’t eat. F3

8) I feel pride when others compliment the control I have in avoiding eating. F3

9) The thinner I get, the more proud I am of myself. F1

10) I have a sense of pride when I lose weight. F1

11) I feel pride when others notice I have lost weight. F1

12) Starving and not eating make me feel pride. F3

13) I feel pride when I accomplish my body shape targets. F1

14) I feel pride when I succeed in following definite diet rules (e.g., a calorie limited diet, not eating chocolate). F1

15) Limiting my eating when others cannot makes me feel proud. F3

16) Being able to eat regular meals makes me feel pride. F2

17) I would feel proud of myself if I lost more weight. F1

18) I feel pride when I manage to control my weight. F1

19) I would feel more pride if I became skinnier. F1

20) When other people comment on my extreme thinness, I feel a sense of pride. F4

21) Not eating over long periods of time (despite feeling hungry) makes me feel pride. F3

22) I feel pride when I manage to lose more weight than I expected. F1

23) I feel a sense of pride if I do not eat sweets, desserts and puddings. F1

24) Being underweight makes me feel pride. F3

25) Eating without worrying about how many calories I am consuming makes me feel pride. F2

26) When people tell me how thin I look I feel a sense of pride. F4

27) I feel pride when I can eat 3 standard-size meals a day without making myself sick (e.g., purging). F2

28) When my weight continues to go down week after week I feel pride. F1

29) I feel pride when I fit into a size of clothing that I aspire to wear (the smaller the better). F1

30) I feel proud of the effort I put into achieving the way I look now. F2

31) Keeping to my diet rules when others cannot makes me feel pride. F3

32) I feel pride when I experience having an empty stomach. F3

33) Limiting the amount of calories I eat per day makes me feel proud. F1

34) I feel pride when I manage to control my body shape. F1

35) I feel pride when I stay hungry. F3

36) I would feel pride if I accomplish my weight aims. F1

37) I experience feelings of pride when others say they don’t know how I manage to stay so lean. F3

38) I feel proud of looking healthy. F2

39) When I weigh myself and I notice I have lost weight I feel pride. F1

40) I would feel pride if my body shape looks the way I want. F1

41) Other people pointing out that I am extremely thin makes me feel a sense of pride. F4

42) If I could lose weight more quickly, I would feel a sense of pride. F1

43) I feel pride when others compliment the control I have over my eating. F3

44) I am proud of eating healthily. F2

45) I feel proud when others compliment my ability to limit how much I eat during the day. F3

46) I would feel proud of myself if I lost weight. F1

47) Being able to limit my food intake while other people cannot makes me feel proud of myself. F3

48) When others compliment me for not eating very much I feel proud. F3

49) I am proud of myself when I accomplish my weight aims. F1

50) Eating less than others gives me a sense of pride. F3

51) I would feel pride if I was thinner. F1

52) I feel proud of maintaining a healthy weight. F2

53) I would feel pride if my weight went down. F1

54) Fasting makes me feel pride. F3

55) I feel pride when others tell me to eat and I refuse to do it. F3

56) Not eating high-fat food when others cannot resist it makes me feel pride. F3

57) I am proud because I have learnt to eat healthily. F2

58) Having a healthy weight makes me feel pride. F2

59) I would feel pride if I was able to eat less. F1

60) I would feel pride if I was a low weight. F1

FI: Pride in weight loss, food control. and thinness; FII. Pride in outperforming others and social recognition. FIII. Pride in healthy weight and healthy eating; FIV: Pride in capturing other people’s attention due to extreme thinness.

**Table A1**

Comparison of means on sociodemographic variables and total PEP-S scores between two random samples of women.

| Variables | Sample 1 (*n*=472)  (*M, SD*) | Sample 2 (*n*=482)  (*M, SD*) | *t* | *gl* | *p* |
| --- | --- | --- | --- | --- | --- |
| Age | 21.68 (2.90) | 21.62 (2.79) | .30 | 952 | .755 |
| SCI | 36.72 (17.82) | 36.49 (17.49) | .984 | .199 | .842 |
| PEPS-Total | 191.24 (80.03) | 188.10 (79.36) | .687 | .608 | .544 |

Note: SCI: Social Class Index (Hollingshead, 1975)

**Table A2**

Factor solution for EFA (women; *n* = 472).

|  | EFA structure matrix | | | |
| --- | --- | --- | --- | --- |
| Items | FI | FII | FIII | FIV |
| PEPS1 | 0.999 |  |  |  |
| PEPS4 | 0.999 |  |  |  |
| PEPS19 | 0.998 |  |  |  |
| PEPS5 | 0.997 |  |  |  |
| PEPS46 | 0.997 |  |  |  |
| PEPS53 | 0.996 |  |  |  |
| PEPS17 | 0.973 |  |  |  |
| PEPS51 | 0.971 |  |  |  |
| PEPS39 | 0.957 |  |  |  |
| PEPS10 | 0.954 |  |  |  |
| PEPS22 | 0.948 |  |  |  |
| PEPS42 | 0.919 |  |  |  |
| PEPS11 | 0.899 |  |  |  |
| PEPS28 | 0.861 |  |  |  |
| PEPS60 | 0.856 |  |  |  |
| PEPS9 | 0.841 |  |  |  |
| PEPS26 | 0.830 |  |  |  |
| PEPS29 | 0.758 |  |  |  |
| PEPS36 | 0.592 |  |  |  |
| PEPS59 | 0.591 |  |  |  |
| PEPS18 | 0.542 |  |  |  |
| PEPS40 | 0.493 |  |  |  |
| PEPS49 | 0.482 |  |  |  |
| PEPS25 | 0.426 |  |  |  |
| PEPS47 |  | 0.978 |  |  |
| PEPS35 |  | 0.962 |  |  |
| PEPS32 |  | 0.958 |  |  |
| PEPS55 |  | 0.954 |  |  |
| PEPS56 |  | 0.933 |  |  |
| PEPS50 |  | 0.906 |  |  |
| PEPS43 |  | 0.882 |  |  |
| PEPS21 |  | 0.878 |  |  |
| PEPS33 |  | 0.819 |  |  |
| PEPS12 |  | 0.815 |  |  |
| PEPS15 |  | 0.811 |  |  |
| PEPS54 |  | 0.807 |  |  |
| PEPS48 |  | 0.804 |  |  |
| PEPS45 |  | 0.767 |  |  |
| PEPS7 |  | 0.747 |  |  |
| PEPS31 |  | 0.744 |  |  |
| PEPS8 |  | 0.732 |  |  |
| PEPS23 |  | 0.721 |  |  |
| PEPS37 |  | 0.661 |  |  |
| PEPS41 |  | 0.627 |  |  |
| PEPS6 |  | 0.621 |  |  |
| PEPS14 |  | 0.519 |  |  |
| PEPS20 |  | 0.509 |  |  |
| PEPS58 |  |  | 0.753 |  |
| PEPS57 |  |  | 0.749 |  |
| PEPS44 |  |  | 0.741 |  |
| PEPS38 |  |  | 0.623 |  |
| PEPS52 |  |  | 0.605 |  |
| PEPS16 |  |  | 0.510 |  |
| PEPS34 |  |  | 0.440 |  |
| PEPS30 |  |  | 0.415 |  |
| PEPS13 |  |  | 0.368 |  |
| PEPS27 |  |  | 0.350 |  |
| PEPS2 |  |  |  | 0.828 |
| PEPS3 |  |  |  | 0.730 |
| α ordinal | 0.984 | 0.973 | 0.757 |  |
| % Variance explained and eigenvalue | 57.83% (34.698) | 9.69% (5.814) | 4.87%  (2.925) | 2.02%  (1.214) |
| FII | .857 |  |  |  |
| FIII | .441 | .336 |  |  |
| FIV | .239 | .166 | .285 |  |

Note: loadings < 0.30 were omitted. FI: Pride in weight loss, food control. and thinness; FII. Pride in outperforming others and social recognition. FIII. Pride in healthy weight and healthy eating; FIV: Pride in capturing other people’s attention due to extreme thinness.
